# SE-MTCAELoc: SE-Aided Multi-Task Convolutional Autoencoder for Indoor Localization with Wi-Fi

**DOI:** 10.3390/s26030945

**Published:** 2026-02-02

**Authors:** Yongfeng Li, Juan Huang, Yuan Yao, Binghua Su

**Affiliations:** 1 Faculty of Data Science, City University of Macau, Macau, China; d22092100211@cityu.edu.mo (Y.L.);; 2 Beijing Institute of Technology, Zhuhai 519088, China; 3 Key Laboratory of Short-Range Radio Equipment Testing and Evaluation, Ministry of Industry and Information Technology, Zhuhai 519088, China

**Keywords:** indoor localization, WiFi fingerprint, convolutional autoencoder, SE attention mechanism, multi-task learning, RSSI

## Abstract

Indoor localization finds wide-ranging applications in user navigation and intelligent building systems. Nevertheless, signal interference within complex indoor environments and challenges regarding localization generalization in multi-building and multi-floor scenarios have restricted the performance of traditional localization methods based on Wi-Fi fingerprinting. To tackle these issues, this paper presents the SE-MTCAELoc model, a multi-task convolutional autoencoder approach that integrates a squeeze-excitation (SE) attention mechanism for indoor positioning. Firstly, the method preprocesses Wi-Fi Received Signal Strength (RSSI) data. In the UJIIndoorLoc dataset, the 520-dimensional RSSI features are extended to 576 dimensions and reshaped into a 24 × 24 matrix. Meanwhile, Gaussian noise is introduced to enhance the robustness of the data. Subsequently, an integrated SE module combined with a convolutional autoencoder (CAE) is constructed. This module aggregates channel spatial information through squeezing operations and learns channel weights via excitation operations. It dynamically enhances key positioning features and suppresses noise. Finally, a multi-task learning architecture based on the SE-CAE encoder is established to jointly optimize building classification, floor classification, and coordinate regression tasks. Priority balancing is achieved using weighted losses (0.1 for building classification, 0.2 for floor classification, and 0.7 for coordinate regression). Experimental results on the UJIIndoorLoc dataset indicate that the accuracy of building classification reaches 99.57%, the accuracy of floor classification is 98.57%, and the mean absolute error (MAE) for coordinate regression is 5.23 m. Furthermore, the model demonstrates exceptional time efficiency. The cumulative training duration (including SE-CAE pre-training) is merely 9.83 min, with single-sample inference taking only 0.347 milliseconds, fully meeting the requirements of real-time indoor localization applications. On the TUT2018 dataset, the floor classification accuracy attains 98.13%, with an MAE of 6.16 m. These results suggest that the SE-MTCAELoc model can effectively enhance the localization accuracy and generalization ability in complex indoor scenarios and meet the localization requirements of multiple scenarios.

## 1. Introduction

Scholars have long delved into the concept of precise localization. The discovery of radio waves in the late 19th century established the groundwork for radio-based navigation and localization systems [1]. Location determination can be categorized into three types: outdoor localization, indoor localization, and seamless indoor–outdoor localization [2]. Among these, indoor localization faces particular challenges due to the presence of indoor obstacles and complex environments, especially when dealing with multi-building and multi-floor indoor scenarios.

Indoor localization finds wide applications in user and robot localization and navigation, manufacturing facilities, large warehouses, smart homes, intelligent factories and buildings, healthcare, intelligent greenhouses, and related domains  [3,4]. In 2020, the global market value of indoor positioning and navigation reached 6.1 billion US dollars, and it is predicted to reach 17 billion US dollars by 2025 [5].

Indoor localization methods based on Wi-Fi can be primarily categorized into distance-based localization and fingerprint-based localization. Distance-based approaches (e.g., triangulation and trilateration) rely on parameters such as Time of Arrival (TOA) and Angle of Arrival (AOA) to compute distances or angles. Nevertheless, these methods exhibit a high degree of dependence on infrastructure, necessitating specialized hardware and prior knowledge of Access Point (AP) positions. This results in substantial deployment costs and limited scalability, rendering large-scale promotion arduous [6].

In contrast, fingerprint-based localization technology has emerged as a research focal point in the domain of indoor positioning. This is primarily attributed to its core advantage of leveraging the existing Wi-Fi infrastructure without the need for additional hardware  [7]. In this context, the term “fingerprint” specifically refers to the unique combination of physical-layer features at a specific physical location. Depending on the sensing approach and application context, these features may include electrical signals, magnetic fields, acoustic signals, or visible-light intensity [8]. Among them, Wi-Fi-based fingerprinting (the most widely adopted form) generally relies on two critical indicators: RSSI and Channel State Information (CSI) [9,10].

The implementation of this technology consists of two core stages. Firstly, offline fingerprint library construction involves collecting RSSI signals from surrounding Access Points (APs) at reference points within the target area and simultaneously recording the corresponding geographical location information (such as building/floor labels and coordinates) to establish a one-to-one mapping database of “signal features-physical positions”. Secondly, online real-time localization involves the positioning terminal collecting real-time RSSI features at its current location and determining its position through matching calculations with the offline fingerprint library.

Its theoretical foundation lies in the spatial uniqueness of signal propagation. Environmental factors, including path loss, multipath effects, and obstacle occlusion, result in differentiated distribution patterns of RSSI signals at various locations, which provides a fundamental guarantee for the discriminability of fingerprints.

Among diverse indoor positioning technologies, Wi-Fi technology has consistently been a favored option owing to its simple deployment and cost-effectiveness. Specifically, fingerprint-based localization not only preserves the aforementioned advantages but also exploits the spatial uniqueness of signals. As a result, it emerges as a highly promising method for complex multi-building and multi-floor scenarios.

RSSI is distinguished by a comparatively low acquisition threshold, which allows for direct collection via standard Wi-Fi networks. It exhibits the advantages of low deployment costs and high portability, which is in full accordance with the core advantage of reusing existing infrastructure. Conversely, CSI attains centimeter-level positioning through its distinctive capabilities, including capturing phase–amplitude details in multipath propagation, adapting to non-line-of-sight (NLOS) environments, and enhancing fingerprint information through spatial/frequency diversity. Nevertheless, CSI necessitates more intricate data preprocessing, higher hardware specifications, and incurs greater computational overhead owing to its bandwidth-dependent processing [8,10,11].

Nevertheless, considering the vulnerability of Wi-Fi RSSI signals to environmental interference and their limited generalization across multiple scenarios, the advent of machine learning and deep learning has provided novel research directions for Wi-Fi indoor positioning [7].

Beyond signal interference and limited generalization ability, a critical practical challenge for fingerprint-based localization pertains to calibration cost. Conventional methods necessitate laborious sampling at reference points for the initial establishment of the database, along with frequent re-calibration to tackle signal drift [3]. Existing strategies to mitigate this burden include semi-supervised learning [12], crowdsourced updates [13], transfer learning [14], and CSI-assisted stability improvement [1,15]. Notably, multi-source data fusion has emerged as a promising approach. The integration of a Wi-Fi received signal strength indicator (RSSI) with low-cost auxiliary data (e.g., inertial measurement unit (IMU) gait data [16], smartphone magnetometer/accelerometer data, or CSI features [17]) can complement discrete fingerprint information, reduce the requirements for reference point density, and prolong the validity of fingerprints. For instance, the continuous motion trajectories provided by IMU decrease the need for dense RSSI sampling during the initial calibration, while the stability of CSI alleviates RSSI drift and reduces the frequency of re-calibration. Our research contributes to this area by enhancing the robustness and generalization of RSSI fingerprints, thus reducing the demand for frequent re-calibration in dynamic environments.

Wi-Fi-based indoor localization deep learning models typically involve two phases: offline training and online prediction. In addition to offline learning, deep learning can also be used for feature extraction. In multi-building and multi-floor environments, the primary objectives of prediction are to classify buildings and floors, as well as perform location regression [18].

Limitations in Feature Extraction of Traditional Autoencoders (AE/SAE): In 2017, the stacked autoencoder (SAE) proposed by Nowicki et al. attained a 92% accuracy rate in building/floor classification. Nevertheless, its one-dimensional feature input was unable to capture the spatial correlation information from the RSSI, and it overlooked the influence of environmental noise on feature quality, thereby resulting in suboptimal generalization performance in multi-floor complex scenarios [19]. In 2018, the extended SAE model developed by Kim et al., although capable of supporting location regression, lacked an architecture designed for the “coordinated optimization of classification and regression tasks,” which led to performance conflicts between the two tasks (wherein higher classification accuracy was associated with increased regression errors) [20].

Deficiencies in Feature Weight Allocation of Convolutional Neural Network (CNN/CAE): In 2019, Zhao et al. [21] were the first to utilize CAE, and in 2021, Qin et al. [22] introduced the CDAE-CNN model. Although these models enhanced spatial feature extraction through two-dimensional transformations, their “equalized channel weighting” mechanism was incapable of dynamically assigning priority to crucial signal channels (e.g., strong RSSI signals from specific APs) or mitigating environmental interference (e.g., weak noise caused by wall obstructions). This shortcoming resulted in the classification accuracy remaining below 99% in multi-building scenarios. In 2024, the CNN-CAE model proposed by Kargar-Barzi et al. [23] streamlined the localization process; however, it converted regression tasks into classification tasks, sacrificing fine-grained coordinate precision and failing to meet the real-world requirements for continuous positioning.

The absence or imbalance of multi-task learning architectures: Most existing models (e.g., WiFiNet [24]) focus solely on single tasks (classification or regression). Few multi-task models (e.g., HADNN [18]) lack reasonable loss weight allocation strategies, resulting in excessively high errors for core localization tasks (coordinate regression) (average absolute error MAE generally >9 m). This imbalance fails to balance the dual requirements of “building/floor classification accuracy” and “coordinate regression precision”.

To tackle the aforementioned three categories of deficiencies (inadequate feature extraction, inappropriate feature weighting, and imbalanced multi-task optimization), this paper puts forward a multi-task CAE model incorporating a squeeze-excitation (SE) attention mechanism. The specific solutions are customized as follows:

(1) The one-dimensional RSSI signal is transformed into a 24 × 24 two-dimensional matrix and fed into the CAE. By leveraging local receptive fields within the convolutional layers to uncover spatial correlation information, and introducing Gaussian noise enhancement (with a mean of 0 and a standard deviation of 5) during data preprocessing, the model’s noise resistance is improved. This mitigates the limitation of traditional AE models that solely rely on one-dimensional features.

(2) An SE attention mechanism module is introduced, which dynamically learns feature channel weights through a “squeeze-excite” operation (e.g., assigning high weights to AP signal channels with strong localization associations and low weights to noise channels). Experimental results suggest that this module reduces the Mean Squared Error (MSE) of the CAE pre-trained model by 26.2% and the MAE by 14.2% on validation sets, effectively enhancing key features and suppressing interference.

(3) A three-task integrated architecture is constructed, combining building classification, floor classification, and coordinate regression. A weighted loss strategy is adopted (with weights of 0.1 for building classification, 0.2 for floor classification, and 0.7 for coordinate regression). By exploiting scene constraints from classification tasks (such as the signal distribution features of “a specific building’s floor”), the accuracy of the regression task is optimized. On the UJIIndoorLoc dataset, this approach achieves a building classification accuracy of 99.57% and a floor classification accuracy of 98.57%, while reducing the MAE of coordinate regression to 5.23 m, attaining balanced multi-task performance.

The subsequent sections of this paper are organized as follows: In Section 2, a systematic review of relevant research findings in the field of indoor positioning technology will be conducted, with an emphasis on analyzing the current research progress and technical challenges. Section 3 provides a comprehensive elaboration of the methodological framework proposed by our research group. Section 4 explores the core components of the model and their optimization strategies through experimental studies. Finally, Section 5 concludes the entire paper.

## 2. Related Work

An autoencoder, a type of neural network, is predominantly utilized for feature extraction, dimensionality reduction, and data compression [25]. This technology was initially applied in indoor positioning by Nowicki et al. [19] in 2017. On the UJIIndoorLoc dataset, researchers adopted a stacked autoencoder (SAE) architecture with a (256-128-64) structure, combined with a classification framework, attaining an accuracy of 92% in building/floor classification. Subsequently, in 2018, Kim et al. [20] extended the SAE technology to multi-building and multi-floor indoor positioning tasks, effectively realizing the dual functions of building/floor classification and position regression.

When employing CNNs for indoor localization, it is generally requisite to transform one-dimensional RSSI values into two-dimensional data [26,27]. A 13-layer convolutional network, namely WiFiNet [24], was developed. This network first transforms the RSSI values of Wi-Fi APs into images before inputting them into the CNN network. In 2024, Arslantas et al. proposed a model architecture that performs joint training of an autoencoder and a CNN to address issues such as sparse Wi-Fi fingerprint data and difficulties in feature extraction [28]. This model utilizes an autoencoder to optimize Wi-Fi fingerprints before inputting them into a CNN for classification and regression, thus achieving the dual objectives of floor classification and coordinate localization.

CAEs integrate the unsupervised learning characteristics of autoencoders (AEs) with the spatial learning capabilities of CNNs [29]. In 2019, Zhao et al. [21] first applied CAEs in the field of indoor positioning, and designed a three-layer CAE architecture. Subsequently, in 2021, Qin et al. [22] proposed CCpos, a Wi-Fi fingerprint indoor positioning system, which combines Convolutional Denoising Autoencoders (CDAE) with CNNs. The system first purifies the Received Signal Strength Indicator (RSSI) signals via CDAE to extract robust features, and then uses CNNs to determine the positioning results. By 2024, Kargar-Barzi et al. [23] put forward a simplified indoor positioning method that combines CNNs with convolutional autoencoders. This method adopts a regional grid strategy to transform regression-based positioning into classification tasks, thus achieving improved classification accuracy.

In recent years, attention mechanisms have been widely employed in the field of indoor localization. By dynamically focusing on key features and suppressing redundant information, this mechanism effectively addresses issues such as signal noise and multi-modal heterogeneity present in complex indoor environments, thereby significantly improving positioning accuracy and robustness. In the research on RSSI fingerprint positioning for LoRaWAN networks [30], researchers enhanced CNN models using Squeeze-Excitation (SE) blocks (a variant of channel attention). Through the “squeeze-excite” process, the model strengthens the gateway RSSI channel features that contribute more to positioning.

In the research on indoor scene recognition-assisted positioning [31], the model integrated single self-attention with multi-head attention. Single self-attention was used to extract visual local discriminative features (e.g., objects within scenes), while multi-head attention was utilized to fuse global scene features. In the research on Wi-Fi fingerprint positioning [32], the MSNN-Loc model introduced channel attention and multi-head self-attention. Channel attention was employed to filter invalid Access Point (AP) signals and amplify stable AP features, and multi-head self-attention was applied to capture the global spatial correlations of AP signals. In the research on Wi-Fi and Inertial Measurement Unit (IMU) fusion positioning [16], the DbDIO model adopted temporal attention (TA) and branch attention. Temporal attention focused on effective IMU motion time segments (e.g., gait peaks), and branch attention was used to fuse multi-scale motion features.

This paper converts Wi-Fi RSSI into two-dimensional data, which is subsequently directly inputted into a CAE. The CAE integrates the SE attention mechanism. Through the utilization of a multi-task framework, this study concurrently achieves the classification of buildings and floors, as well as coordinate regression, especially in the context of architectural environments.

A comparative analysis of the relevant literature is presented in Table 1.

## 3. Proposed Method

**Figure 1:**
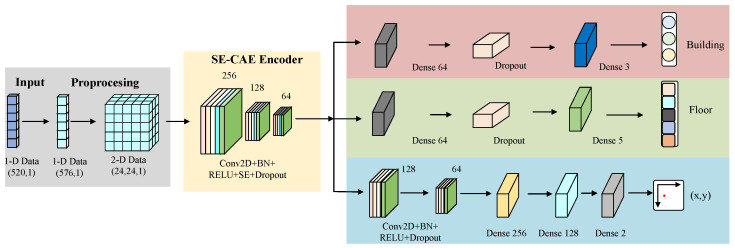
Framework diagram of the proposed model.

This paper puts forward an indoor localization model which employs a two-dimensional CAE integrated with attention mechanisms for CNN-based triple-task learning. Through the conversion of Wi-Fi signal features into two-dimensional image data, the model attains accurate positioning across multiple buildings and floors. Figure 1 depicts the framework of this multi-task structural localization model, and detailed explications of its components are presented below.

### 3.1. Network Structure

The SE-MTCAELoc network is a multi-task indoor positioning algorithm that relies on RSSI signals. Its core procedure is “data preprocessing → pre-training of the SE convolutional autoencoder → fine-tuning of the multi-task model,” as depicted in Figure 1. Initially, the original 520-dimensional RSSI signals are subjected to Gaussian noise enhancement, 24 × 24 matrix padding, and label encoding/normalization operations. Subsequently, the SE convolutional autoencoder is trained (the encoder compresses features into a 6 × 6 × 64 bottleneck feature via three layers of convolution, with each layer integrating an SE module for dynamic channel weighting, and the decoder symmetrically reconstructs the input, accomplishing unsupervised pre-training with MSE loss). Subsequently, a multi-task model is built based on the pre-trained encoder.

The multi-task head of the SE-MTCAELoc network constructed based on the 6×6×64 bottleneck feature output by the shared encoder, designing differentiated branches for three types of tasks: These tasks exhibit fundamental disparities in positioning objectives, network architectures, optimization priorities, and evaluation criteria to accommodate hierarchical localization requirements.

Specifically, the building classification head compresses features into a 64-dimensional vector through global average pooling, processed by a fully connected layer of 64 neurons (with ReLU activation and $L_{2}$ regularization) and dropout (0.3), and outputs the probability distribution of three building categories through a 3-neuron Softmax layer. It functions as coarse-grained scene identification to offer top-level spatial constraints, which is evaluated by accuracy and F1-score with a loss weight of 0.1; the floor classification head utilizes the same pooling and fully connected structure, completing five-floor classification through a 5-neuron Softmax layer, and it achieves medium-grained layer division to narrow the positioning scope, employing the same evaluation metrics as those used in building classification, yet with a higher loss weight of 0.2; the coordinate regression head retains spatial features, first enhanced by 128 3×3 and 64 3×3 convolution kernels (all with ReLU activation and $L_{2}$ regularization), then flattened and processed by a fully connected layer of 256 neurons (with ReLU, batch normalization), and dropout (0.3), followed by a fully connected layer of 128 neurons with ReLU and dropout (0.3), and finally outputs normalized (x,y) coordinates through a 2-neuron linear layer. As the fine-grained core positioning task, it is evaluated by mean absolute error (MAE) and assigned the highest loss weight of 0.7 to prioritize positioning precision.

In consideration of the characteristics of the two datasets, UJIIndoorLoc and TUT2018, utilized in this study, the regression algorithm is adjusted deferentially. Specifically, UJIIndoorLoc requires three-level constraints of building-floor-coordinates. By concatenating the embedding vectors of the building/floor classification results with the bottleneck features, the prediction scope is restricted (e.g., confining the signal distribution scope of a specific building–floor combination). In contrast, TUT2018 only includes a single building; therefore, the classification branch is eliminated, and the coordinate scope is directly constrained by the floor classification results (3 floors). The SE module is optimized to capture the signal features related to direction, thereby improving the positioning accuracy in fine-grained scenarios.

The three task heads are jointly optimized through weighted loss (building 0.1, floor 0.2, coordinates 0.7), achieving task-specific learning on shared features, balancing the differences between classification and regression characteristics to enhance overall positioning performance. This weight configuration is determined via systematic comparative experiments and task priority analysis: (1) Coordinate regression functions as the core task of indoor positioning; therefore, it is assigned the highest weight to ensure the accuracy of core localization. (2) Building and floor classification provide scene constraints for regression (e.g., the signal distribution patterns of specific building–floor combinations), and their weights are set to maintain high classification accuracy while preventing competition with regression for optimization resources. (3) Comparative experiments verify that this configuration avoids performance conflicts among tasks and achieves the optimal trade-off between classification accuracy and regression precision. Detailed experimental validation is presented in Section 4.7. The SE module significantly improves the accuracy of fine-grained positioning tasks by enhancing key signal channel features.

### 3.2. SE-CAE

**Figure 2:**
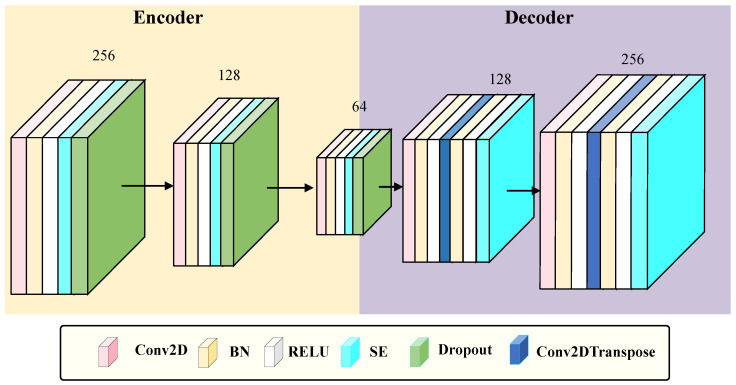
The architectural configuration of the Squeeze-and-Excitation Convolutional Autoencoder (SE-CAE).

As illustrated in Figure 2, the SE-CAE belongs to a category of CAE that integrates the SE attention mechanism [30,37]. Its input is an RSSI signal matrix with dimensions of 24×24×1, which is obtained from preprocessing procedures involving padding and reshaping of raw RSSI features. The primary aim of the SE-CAE is to acquire robust and noise-resistant feature representations of RSSI signals through an unsupervised reconstruction task, and simultaneously enhance discriminative channel features dynamically via the SE module.

Let the preprocessed RSSI 2D matrix be defined as the input feature map of the SE-CAE, which is mathematically expressed as:

(1) $$X\in R^{H\times W\times C_{\mathrm{in}}}$$

where H=24 and W=24 represent the height and width of the feature map, corresponding to the 24 × 24 matrix shape obtained by padding the original 520-dimensional RSSI features to 576 dimensions; $C_{\mathrm{in}}=1$ denotes the number of input channels, as the RSSI signal is treated as a single-channel feature map to fit the input requirement of the convolutional layer.

#### 3.2.1. The Encoder of SE-CAE

The encoder compresses the features into a bottleneck feature of size 6×6×64 through three layers of convolution operations (utilizing 2563×3, 1283×3, and 643×3 convolution kernels, all equipped with $L_{2}$ regularization (λ=0.001), Batch Normalization (BN), ReLU activation, and Dropout (0.3)), in combination with MaxPooling2D. After each convolution layer, an SE module is inserted (spatial information is compressed by means of Global Average Pooling 2D, channel weights are learned via a fully connected layer with a reduction factor of 16, and the weights are output through the sigmoid function and multiplied by the input features).

Each convolution block uses 3×3 convolution kernels with stride s=1 and padding p=1 to extract local features without reducing spatial dimensions. For the *k*-th convolution block, the operation is defined as:

(2) $${\mathrm{Conv}}_{k}\left(Z_{k-1}\right)=\sum_{i=1}^{3}\sum_{j=1}^{3}Z_{k-1}\left[:,:,:\right]*W_{k}\left[i,j,:,:\right]+b_{k}$$

where $Z_{k-1}$ is the input feature map of the *k*-th block, $W_{k}\in R^{3\times 3\times C_{\mathrm{in},k}\times K_{k}}$ is the convolution kernel matrix, $b_{k}\in R^{K_{k}}$ is the bias term, $C_{\mathrm{in},k}$ is the number of input channels, $K_{k}$ is the number of convolution kernels, and ∗ denotes element-wise multiplication.

Block 1: $C_{\mathrm{in},1}=1$, $K_{1}=256$, output shape =24×24×256.

Block 2: $C_{\mathrm{in},2}=256$, $K_{2}=128$, output shape =12×12×128 (followed by 2×2 MaxPooling2D with stride 2).

Block 3 (Bottleneck Layer): $C_{\mathrm{in},3}=128$, $K_{3}=64$, output shape =6×6×64 (followed by 2×2 MaxPooling2D with stride 2).

BN standardizes the feature distribution to accelerate training convergence and reduce overfitting:

(3) $$\mathrm{BN}\left(Y\right)=\gamma \cdot \frac{Y-\mu_{B}}{\sqrt{\sigma_{B}^{2}+\epsilon }}+\beta$$

where $\mu_{B}=\frac{1}{N}\sum_{n=1}^{N}Y_{n}$ and $\sigma_{B}^{2}=\frac{1}{N}\sum_{n=1}^{N}{\left(Y_{n}-\mu_{B}\right)}^{2}$ are the mean and variance of the mini-batch, γ and β are learnable scaling and shifting parameters, and $\epsilon =10^{-5}$ to avoid division by zero.

ReLU introduces non-linearity to model complex patterns in RSSI signals:

(4) ReLU(Z)=max(0,Z).

The SE module enhances the discriminative features and suppresses noise through the dynamic adjustment of channel weights, as elaborated in Section 3.2.1.

A dropout rate of 0.3 is used to randomly deactivate neurons, preventing overfitting:

(5) $$\mathrm{Dropout}\left(Z\right)=Z\cdot M,\;M_{i,j,c}∼\mathrm{Bernoulli}\left(0.7\right)$$

where M is a binary mask matrix with the same shape as Z.

MaxPooling2D reduces spatial dimensions while preserving key feature information:

(6) $$\mathrm{MaxPool}\left(Z\right)=\max_{\begin{array}{c} i=1,2;\\ j=1,2 \end{array}}Z\left[h+i-1,\;w+j-1,\;c\right]$$

for each spatial position (h,w) and channel *c*.

After three convolution blocks, the encoder outputs the bottleneck feature $E\in R^{6\times 6\times 64}$, which is a compact representation of the original RSSI signal.

#### 3.2.2. The Decoder of SE-CAE

The decoder symmetrically reconstructs the bottleneck features into an output of 24×24×1 through convolutional layers and transposed convolutional layers (employing 1282×2 and 2562×2 transposed convolution kernels, with a stride of 2), also inserting SE modules. Ultimately, the reconstruction of the input signal is achieved by minimizing the Mean Squared Error (MSE) loss. This method preserves the spatial feature extraction ability of the convolutional autoencoder while dynamically enhancing key channel features through the SE module, thereby enhancing the robustness against noisy signals.

Block 1: Uses 128×2×2 transposed convolution kernels with stride 2 to upsample E from 6×6×64 to 12×12×128.

Block 2: Uses 256×2×2 transposed convolution kernels with stride 2 to upsample to 24×24×256.

Final Conv2D: Uses a 3×3 convolution kernel to map the multi-channel feature map to a single-channel output $\hat{X}$:

(7) $$\hat{X}=\mathrm{Conv}2D\left({\mathrm{Dec}}_{2},\;W_{\mathrm{final}},\;\mathrm{padding}=‘\mathrm{same}’\right)+b_{\mathrm{final}}$$

where ${\mathrm{Dec}}_{2}$ is the output of the second decoder block, $W_{\mathrm{final}}\in R^{3\times 3\times 256\times 1}$ is the final convolution kernel, and $b_{\mathrm{final}}\in R^{1}$ is the corresponding bias.

The SE-CAE is pretrained with the goal of minimizing the Mean Squared Error (MSE) between the input X and the reconstructed output $\hat{X}$:

(8) $$L_{CAE}=\frac{1}{H\times W\times C_{\mathrm{in}}}\sum_{h=1}^{H}\sum_{w=1}^{W}\sum_{c=1}^{C_{\mathrm{in}}}{\left(X\left[h,w,c\right]-\hat{X}\left[h,w,c\right]\right)}^{2}$$

This loss function drives the network to learn the intrinsic structure of RSSI signals and generate noise-resistant feature representations.

### 3.3. SE Attention Mechanism Module

In the proposed network, the attention mechanism employs the SE module [37]. The core function of this module is to enhance significant features and suppress redundant information by learning the importance weights of feature channels. The workflow can be divided into three steps, as shown in Algorithm 1.
**Algorithm 1** Workflow of SE Attention Mechanism**Require:** 
  input_feature_map: Feature map output from the convolutional layer in SE-CAE, with dimension (H,W,C) (where H=24, W=24; C=256,128,64 corresponding to the number of channels of the three convolutional layers in the encoder );    reduction_ratio: Channel reduction ratio, default value = 16;**Ensure:** 
  weighted_feature_map: Feature map after dynamic channel weighting, with the same dimension (H,W,C) as the input, used as the input to the next layer of SE-CAE;1:**Squeeze Operation: Aggregate Spatial Information**2:Extract *H* (height), *W* (width), and *C* (channel number) from input_feature_map;3:Perform Global Average Pooling 2D on each channel to convert input_feature_map from (H,W,C) to (1,1,C);4:Reshape the result into a 1D vector squeezed_vector with dimension (C,);5:**Excitation Operation: Learn Channel Weights**6:Construct the first dense layer: take squeezed_vector as input, set neurons to C//reduction_ratio, use ReLU activation;7:Construct the second dense layer: take the output of the first dense layer as input, set neurons back to *C*, use Sigmoid activation to get channel_weights (dimension (C,), range [0, 1]);8:**Weight Application: Dynamic Channel Weighting**9:Reshape channel_weights from (C,) to (1,1,C) to match input_feature_map dimension;10:Perform element-wise multiplication between input_feature_map and reshaped channel_weights to obtain weighted_feature_map;11:**Output Result**12:Return weighted_feature_map as input to the next convolutional layer in SE-CAE;

Firstly, global average pooling compresses the input feature maps (e.g., 24 × 24 × 256) via the squeezing operation. This process aggregates the spatial information of each channel into a single value, thus forming one-dimensional vectors (e.g., 256-dimensional).

Secondly, two layers of dense networks execute the excitation process. The number of channels is reduced to 1/16 (e.g., 256 → 16) to mitigate the computational burden. After ReLU activation, the number of channels is restored to the original level (e.g., 16 → 256).

Finally, Sigmoid activation outputs the channel weights within the range of 0 to 1. Subsequently, the learned weights are multiplied with the original feature maps on a channel-by-channel basis to realize adaptive weighting of features in different channels.

The squeeze operation compresses the spatial dimensions of each channel into a single scalar, capturing the global information of the channel. For an input feature map $F\in R^{H\times W\times C}$ (where H=24,W=24, and *C* denotes the number of channels, e.g., 256, 128, 64 for the three convolutional layers in the encoder), the squeeze vector $s\in R^{C}$ is computed via Global Average Pooling (GAP):

(9) $$s\left(c\right)=\frac{1}{H\cdot W}\sum_{i=1}^{H}\sum_{j=1}^{W}F\left(i,j,c\right)\;\forall c\in \left\{1,2,\ldots ,C\right\}$$

where s(c) represents the global average response of the *c*-th channel, reflecting the overall importance of the channel’s spatial features.

The excitation operation learns the importance of each channel through a two-layer fully connected (FC) network, enabling adaptive weighting to emphasize key channels and suppress noise. Mathematically:

(10) $$e=\sigma_{2}\left(W_{2}\cdot \sigma_{1}\left(W_{1}\cdot s+b_{1}\right)+b_{2}\right)$$

where $W_{1}\in R^{(C/r)\times C}$ is the weight matrix of the first FC layer, with a reduction ratio r=16 (reducing channels from *C* to C/r to reduce computational complexity); $W_{2}\in R^{C\times (C/r)}$ is the weight matrix of the second FC layer, restoring channels to the original dimension *C*. $b_{1}\in R^{\left(C/r\right)}$ and $b_{2}\in R^{C}$ are the bias terms of the two FC layers; $\sigma_{1}\left(\cdot \right)$ denotes the ReLU activation function ($\sigma_{1}\left(x\right)=\max\left(0,x\right)$), introducing non-linearity to model complex channel dependencies; $\sigma_{2}\left(\cdot \right)$ is the Sigmoid activation function ($\sigma_{2}\left(x\right)=\frac{1}{1+e^{-x}}$), constraining the output weights to the range [0,1]; $e\in R^{C}$ is the learned channel weight vector, where e(c) indicates the importance of the *c*-th channel.

The learned weights are applied to the original feature map via element-wise multiplication, dynamically enhancing key channels (e.g., strong RSSI signals from location-relevant APs) and suppressing interference channels (e.g., noise from wall obstructions). The mathematical expression is:

(11) $$F_{\mathrm{weighted}}\left(i,j,c\right)=F\left(i,j,c\right)\cdot e\left(c\right)\;\forall \left(i,j,c\right)$$

where $F_{\mathrm{weighted}}\in R^{H\times W\times C}$ is the output feature map after SE module processing, retaining the same spatial and channel dimensions as the input.

After integrating the SE module into each convolution block, it dynamically regulates the proportion of effective features (e.g., signals highly correlated with location) and interference features (e.g., noise) in RSSI signals. This regulation enhances the model’s sensitivity to critical signals, making it particularly appropriate for scenarios where RSSI signals are prone to interference in complex indoor environments. The SE module’s effectiveness is rooted in its ability to model channel-wise dependencies, and its parameters are optimized jointly with the CAE during pre-training to minimize the reconstruction loss:

(12) $$\min_{\theta_{\mathrm{SE}}}L_{\mathrm{rec}}\left(\mathrm{CAE}(\mathrm{SE}\left(X;\theta_{\mathrm{SE}}\right);\theta_{\mathrm{CAE}})\right)$$

where $\theta_{\mathrm{SE}}=\left\{W_{1},b_{1},W_{2},b_{2}\right\}$ are the trainable parameters of the SE module, $\theta_{\mathrm{CAE}}$ are the parameters of the CAE, and $L_{\mathrm{rec}}$ is the MSE reconstruction loss.

## 4. Experiments and Results

Experiments and validations were carried out on the public dataset UJIIndoorLoc, followed by tests on the TUT2018 dataset. The entire implementation process was grounded in the TensorFlow 2.10.0 framework and accomplished on an RTX 4090 GPU platform.

### 4.1. Dateset

#### 4.1.1. UJIIndoorLoc Dataset

**Figure 3:**
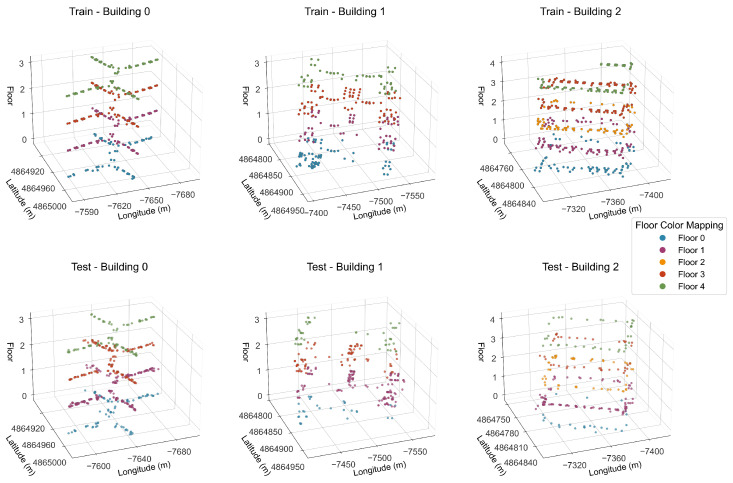
Distribution of Access Points (APs) in Training Set and Test Set on UJIIndoorLoc Dataset.

The UJIIndoorLoc dataset, a widely used open-source dataset in the field of Wi-Fi indoor positioning, is primarily utilized to evaluate the performance of the SE-MTCAELoc model in complex multi-building and multi-floor scenarios. The detailed configuration is presented as follows:

(1) Dataset Scale and Scenario Coverage: As depicted in Figure 3, this dataset encompasses three buildings, each featuring 4 or 5 floors. The training and test sets are composed of 19,937 and 1111 rows of sample data, respectively, accompanied by 529 columns of features. These data cover typical indoor environments, including corridors and offices. Furthermore, data from different time periods are collected to reflect the variations in the stability of environmental signals.

(2) Core Data Fields and Signal Features: The dataset contains 520 Wi-Fi access points (APs) with Received Signal Strength Indicator (RSSI) values, ranging from −104 to 100 dBm. Here, 100 dBm represents invalid signals that need to be replaced during preprocessing. Other components include building labels (with three categories), floor labels (five categories across 14 floors in three buildings), geodetic coordinates (UTM coordinates based on the WGS84 geodetic system for coordinate regression tasks), and timestamps. This comprehensive structure supports three core tasks: building classification, floor classification, and coordinate regression.

(3) Sampling Details: Reference Point (RP) Layout: A total of 933 RPs are meticulously planned, including 1 central RP and 1 corridor entrance RP for each enclosed space (e.g., offices and laboratories) to ensure comprehensive and uniform coverage of internal and transitional areas. The average interval between adjacent RPs falls within the range of 3 to 5 m.

Collection Process: Eighteen participants utilized 16 types of Android devices to collect data through the CaptureLoc application. Each RP was sampled repeatedly by at least two participants to minimize individual biases, and each sampling operation automatically recorded 10 RSSI data points to account for instantaneous signal fluctuations.

Long-Term Stability: Data were collected over diverse time periods to reflect the dynamic changes of environmental signals, thus ensuring the generalizability of the dataset.

(4) Experimental Suitability Statement: The multi-building and multi-floor characteristics of this dataset are highly consistent with the research objective of “multi-task localization” in this paper. It can be used to verify the classification accuracy and regression error of the SE-MTCAELoc model in complex scenarios.

Additionally, its open-access nature facilitates cross-comparisons with existing research (e.g., Kim et al.’s 2018 SAE model and Qin et al.’s 2021 CDAE-CNN model) [20,22] (corresponding to the experimental requirements outlined in Table 1).

#### 4.1.2. TUT2018 Dataset

This dataset is a multi-floor open architectural dataset formulated to assess the generalization ability of the SE-MTCAELoc model across diverse data distributions. The configuration particulars are presented as follows:

(1) Data Scale and Scenario Coverage: It encompasses a single building with three floors (ground, first, and second), consisting of approximately 400 samples. Collected within campus indoor settings, it diverges from UJIIndoorLoc in terms of AP deployment density.

(2) Core Data Fields and Signal Features: It incorporates x/y/z coordinates (where the z-axis corresponds to floor labels), RSSI values of 492 APs (excluding APs with a signal validity of less than 0%), and lacks architectural labels.

(3) Sampling Details: Reference Point (RP) Layout: The reference points are arranged in a dense pattern, with an average spacing of 2 to 3 m, which is in line with the high-precision positioning requirements of the library scenario.

Collection Process: Professional researchers employed Samsung Galaxy S3 devices to collect data. For each reference point, six consecutive RSSI data points were collected in four specific orientations to address signal variations induced by directional obstacles (e.g., bookshelves).

Consistency Control: The same device and collection protocol were adopted throughout the 15-month data collection period to minimize systematic errors.

**Figure 4:**
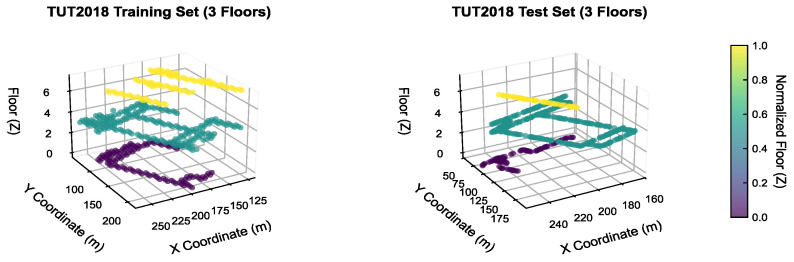
Distribution of Access Points (APs) in Training Set and Test Set on TUT2018 Dataset.

(4) Experimental Adaptability: The three-dimensional coordinate distribution is presented in Figure 4. The training set and test set are uniformly distributed across all floors, and the sampling points encompass typical indoor scenarios, including classrooms, laboratories, and corridors.

#### 4.1.3. Influence of Sampling Parameters on Localization Accuracy

(1) Reference Point Density: The UJIIndoorLoc dataset is distinguished by reference points with a spacing ranging from 3 to 5 m. This research indicates that such a configuration can effectively capture spatial signal variations. Conversely, the TUT2018 dataset, characterized by a denser spacing of 2 to 3 m, is more favorable for fulfilling the fine-grained positioning requirements of libraries. In this study, a floor classification accuracy of 98.13% was attained on the TUT2018 dataset, which validates the effectiveness of high-density reference points in enhancing classification performance.

(2) Sampling Frequency: The UJIIndoorLoc dataset has a sampling frequency of 10 samples per reference point, whereas the TUT2018 dataset has 6 samples per reference point.

(3) Device/Directional Diversity: In the UJIIndoorLoc dataset, 25 devices were employed, and in the TUT2018 dataset, fixed devices with multi-directional acquisition were utilized. These setups demonstrated that signal performance is affected by device models and acquisition directions. For example, in the TUT2018 dataset, there were RSSI differences of 3 to 6 dBm between the orientations facing the bookshelf and the corridor.

This model tackles device heterogeneity through signal normalization during the data preprocessing phase. Simultaneously, the SE module strengthens the direction-dependent signal features to improve the robustness of regression. The single building with diverse AP density configurations validates the architectural flexibility of the model after the removal of architectural classification branches. A comparative analysis with UJIIndoorLoc (e.g., disparities in floor classification accuracy and MAE regression) assesses data adaptability and demonstrates generalization ability.

### 4.2. Data Preprocessing

#### 4.2.1. Based on UJIIndoorLoc Dataset

The data preprocessing procedure of the UJIIndoorLoc dataset is presented in Algorithm 2. Data are loaded from the CSV file, where the initial 520 columns of Received Signal Strength Indicator (RSSI) features are extracted. Columns 520–521 contain latitude and longitude coordinates, column 522 holds floor labels, and column 523 has building labels. To augment the robustness of RSSI features, Gaussian noise with a mean of 0, a standard deviation of 5, and a clipping range between −110 to −30 dBm is incorporated.

**Figure 5:**
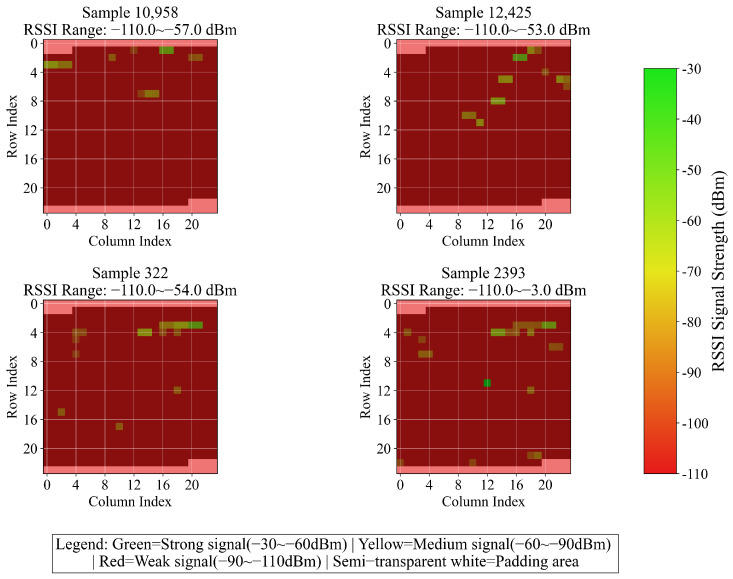
One-dimensional RSSI variation converted into two-dimensional image data (four randomly selected samples).

For compatibility with 2D CNN, the 520 columns of RSSI are padded with −110 dBm to reach 576 elements and then reshaped into a 24 × 24 two-dimensional matrix. Figure 5 presents the variation results of four randomly selected samples. Building and floor labels are initially converted into integers by means of the LabelEncoder, and subsequently transformed into one-hot vectors through the to_categorical function. Coordinates are normalized to the 0–1 range using the MinMaxScaler, and the original ranges and normalization parameters are saved for inverse conversion. Finally, validation is conducted to ensure the accuracy of data processing, and the formatted feature matrices, encoded labels, and metadata are outputted to offer standardized inputs for model training. This methodology preserves crucial information while adapting to the model architecture via enhancement, encoding, and normalization operations, thereby enhancing training stability and laying the groundwork for multi-task learning.

#### 4.2.2. Based on TUT2018 Dataset

In the data preprocessing stage, a comprehensive workflow was formulated, encompassing data loading and cleaning, feature engineering, normalization enhancement, and dataset partitioning. Initially, the CSV data were loaded, and the coordinates of the first three columns (x,y,z) were extracted (where *z* was mapped to three-layer labels of 0/1/2), along with the subsequent RSSI features. Invalid signal values of 100 were replaced with −110 dBm. Subsequently, valid Access Points (APs) were filtered (excluding those with signal effectiveness below 30%), and location-related features were integrated (number of APs with strong signals, signal dynamic range). The original 492 columns of RSSI features were expanded to 576 dimensions by scaling them with −110 dBm. For the training set, augmented samples were generated by applying Gaussian noise (acting only on valid signals within the range of [−100,0]). Meanwhile, the RSSI features were standardized using the StandardScaler, and the (x,y) coordinates were normalized to the range of [0,1] to alleviate scale effects. Finally, the original training and test sets were combined through stratified sampling at an 8:2 ratio, ensuring consistent proportions of samples at the floor level. Floor labels were transformed into one-hot encoding to provide high-quality, balanced, and format-compatible data for subsequent model training.
**Algorithm 2** Preprocessing Flow for UJIIndoorLoc Dataset**Require:** 
Raw UJIIndoorLoc dataset *D* (19,937 samples, 529 features)**Ensure:** 
Standardized feature matrix *X* (24 × 24 × 1), encoded labels *Y* (building/floor), normalized coordinates *C* (x, y)1:**Feature and Label Separation**: Extract the first 520 columns from *D* as RSSI feature matrix *R*, columns 522–523 as floor/building labels *L*, columns 520–521 as original coordinates *P*;2:**RSSI Signal Purification**: Replace invalid signals (value = 100 dBm) in *R* with −110 dBm to get purified matrix $R_{\mathrm{clean}}$;3:**Dimension Adaptation and Enhancement**:4:   a. Pad $R_{\mathrm{clean}}$ to 576 dimensions with −110 dBm, then reshape to 24 × 24 2D matrix $R_{2D}$;5:   b. Generate Gaussian noise matrix *N* (same dimension as $R_{2D}$, mean = 0, std = 5), apply noise to $R_{2D}$, and clip to [−110, −30] dBm to obtain enhanced feature *X*;6:**Label Encoding**: Convert *L* to integer codes, then map to one-hot vectors *Y* (3-dim for buildings, 5-dim for floors);7:**Coordinate Normalization**: Compute $P_{\max}$ and $P_{\min}$ of *P*, normalize *P* to [0, 1] via $C=\left(P-P_{\min}\right)/\left(P_{\max}-P_{\min}\right)$, save $P_{\max}$ and $P_{\min}$ for inverse transformation;8:**Output**: *X*, *Y*, *C*, and normalization parameters ($P_{\max}$, $P_{\min}$);

#### 4.2.3. Fingerprint Uniqueness Evaluation

Wi-Fi fingerprint is defined as a compilation of Received Signal Strength Indicator (RSSI) vectors originating from multiple Access Points (APs). Its distinctiveness lies in the discernible features of vectors across different locations, which constitutes the fundamental premise for localization algorithms.

To conduct a quantitative assessment of the uniqueness of Wi-Fi fingerprints, the average cosine similarity and average Euclidean distance between distinct RSSI vectors are computed. The average cosine similarity serves to gauge the directional correlation among vectors, whereas the average Euclidean distance reflects the numerical disparity. The formulas are defined as follows:

(13) $$\bar{\mathrm{cosine}\_\mathrm{sim}}=\frac{1}{M}\sum_{i=1}^{M}\frac{{\vec{r}}_{i}\cdot {\vec{r}}_{j}}{{∥{\vec{r}}_{i}∥}_{2}\cdot {∥{\vec{r}}_{j}∥}_{2}}$$

(14) $$\bar{\mathrm{euclid}\_\mathrm{dist}}=\frac{1}{M}\sum_{i=1}^{M}\sqrt{\sum_{k=1}^{D}{\left(r_{i,k}-r_{j,k}\right)}^{2}}$$

where M=1000 represents the quantity of sampled RSSI vector pairs, D=576 denotes the dimensionality of the RSSI vector (subsequent to padding to a 24 × 24 matrix and flattening), and ${\vec{r}}_{i},{\vec{r}}_{j}$ signify RSSI vectors originating from distinct physical locations. ${\vec{r}}_{i}\cdot {\vec{r}}_{j}$ denotes the dot product of the two vectors, $∥{\vec{r}}_{i}{∥}_{2}$ and $∥{\vec{r}}_{j}{∥}_{2}$ are the 2-norms of the vectors, and $r_{i,k}$ is the *k*-th component of the *i*-th RSSI vector (i.e., the RSSI value of the *k*-th access point).

Relevant experiments were carried out on the UJIIndoorLoc dataset. The experimental results demonstrated an average cosine similarity of 0.997 (with the preset threshold <0.6) and an average Euclidean distance of 204 dBm (with the preset threshold >15 dBm). Although the cosine similarity value is relatively high, the Euclidean distance is significantly greater than the threshold, indicating that fingerprints from different locations possess distinct discriminability in terms of signal strength. Furthermore, in combination with the enhancement effect of the SE module on key features in the subsequent SE-MTCAELoc model, a coordinate mean absolute error (MAE) of 4.95 m is ultimately achieved, which further validates the effectiveness of the fingerprint library and the model’s feature discrimination ability. The results of the dual-index evaluation jointly suggest that the constructed fingerprint library can provide stable feature support for localization tasks, laying a reliable data foundation for subsequent model optimization.

### 4.3. Pretraining Initialization

SE-CAE pre-training was carried out on RSSI feature data for 100 epochs of unsupervised learning. The training employed the Adam optimizer (with an initial learning rate of 0.001), in conjunction with learning rate decay and early stopping mechanisms. MSE was utilized as the loss function, and MAE was adopted as an auxiliary evaluation metric. The objective was to empower the model to acquire effective feature representations of the RSSI data via the reconstruction task.

**Figure 6:**
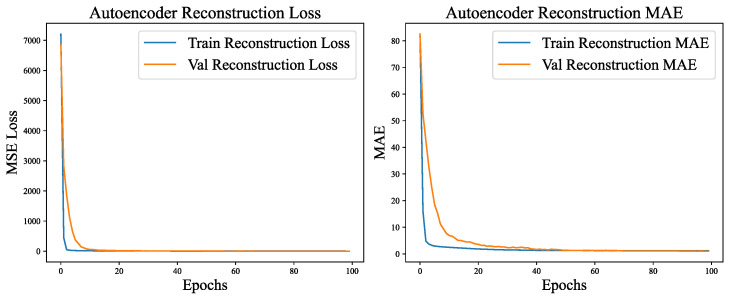
SE-CAE reconstruction loss and MAE curve.

As depicted in Figure 6, the training process manifests distinct three-phase characteristics. In the initial phase (epochs 1–20), the model rapidly assimilated fundamental feature patterns. The MSE declined from 941.42 to 2.39, and the MAE decreased by more than 96%, from 22.78 to 0.89, which illustrates the model’s rapid acquisition of RSSI signal distribution patterns.

The intermediate phase (epochs 21–60) entered a stage of gradual convergence. During this phase, the MSE gradually decreased to 0.68, and the MAE decreased to 0.23. The model started to capture weak signal features and subtle correlations. Despite the slower progress, it maintained a stable decline rate.

In the final phase (epochs 61–100), the model reached a stable state with the learning rates decaying to $6.25\times 10^{-5}$. The MSE of the validation set remained stable at 0.39, while the MAE stabilized at 0.16. The minimal error fluctuations indicate that the model was approaching its optimal state.

The pre-training exhibits remarkable performance and excellent generalization ability. The error differences between the training set and the validation set are minimal (the MSE difference is 0.15, and the MAE difference is 0.03), and no overfitting phenomenon is observed. Remarkably, the error of the validation set is even lower than that of the training set. This can be ascribed to the Gaussian noise introduced in the training set, in contrast to the original data utilized in the validation, which underscores the model’s denoising and reconstruction capabilities. The SE attention module effectively strengthens crucial signal channels during the training process, thereby enhancing the efficiency of feature learning. The final pre-trained weights capture the universal feature representations of the RSSI, offering a high-quality shared encoder foundation for subsequent multi-task localization models. This substantially reduces the training time and enhances the model’s robustness.

During the training process, the model integrated with the SE module demonstrated more rapid convergence in the initial phase, along with a more significant reduction in loss during epochs 5–10. Subsequently, although both models reached a stable state, the model incorporating the SE module consistently sustained lower loss levels. It is worth noting that both models demanded a comparable training time per epoch (all around 3 s), suggesting that the SE module dynamically regulates feature weights via the channel attention mechanism without notably augmenting the computational burden. This mechanism facilitates the enhancement of the extraction of crucial signal features and effectively alleviates noise interference.

In conclusion, the SE attention module dynamically adjusts the weights of RSSI signal channels, enabling autoencoders to concentrate on more critical signal features for localization purposes. This substantially enhances the precision and efficiency of feature reconstruction, laying a foundation for higher-quality feature representation for subsequent multi-task models. It is anticipated to exert a positive influence on downstream tasks such as building classification, floor classification, and coordinate regression.

### 4.4. Attention Mechanism and Its Ablation Experiments

**Figure 7:**
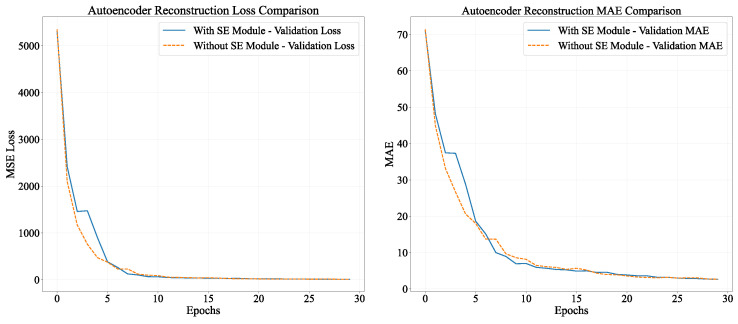
Ablation Experiments on Autoencoder Reconstruction.

In the autoencoder pre-training process, there were significant disparities between models with and without SE modules, as depicted in Figure 7. The final outcomes indicated that the model equipped with SE modules attained a MAE of 5.23 on the validation set. This value was approximately 10.3% lower than the MAE of 5.83 for the model without SE modules, suggesting a remarkable superiority in reconstruction accuracy.

**Figure 8:**
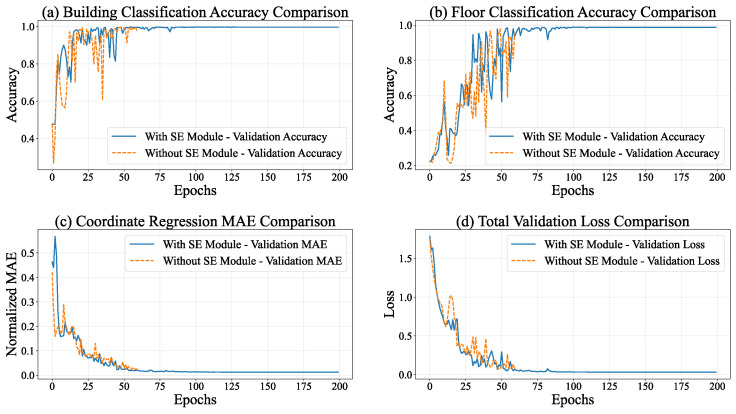
The influence of the SE attention mechanism.

During the training process of the multi-task model equipped with the SE module, the entire model manifested distinct convergence tendencies. As depicted in Figure 8, the performances of all tasks exhibited substantial enhancements. Regarding the total loss, the overall loss of the model declined from 2.0824 at Epoch 1 to 0.0414 at Epoch 80, signifying a reduction exceeding 98%. This phenomenon implies an augmented task-specific fitting capacity, accompanied by refined optimization via adaptive learning rate adjustments (gradually decreasing from 0.001 to $1.56\times 10^{-5}$).

The building classification task demonstrated the most remarkable performance improvement. The training accuracy ascended from 45.74% to 99.64%, while the validation accuracy remained stable at 99.57%. Concurrently, the loss decreased from 1.0747 to 0.0146, indicating the model’s precise recognition of building features. The floor classification task also achieved outstanding results. The training accuracy increased from 23.58% to 98.64%, and the validation accuracy reached 99.58%. Despite certain fluctuations, the loss decreased from 1.5568 to 0.0635, suggesting a strong ability to capture floor features.

In the coordinate regression task, the normalized MAE decreased from 0.5818 at the start of training to 0.0177 on the validation set, corresponding to an error of 5.23 m in actual conversion. Moreover, the loss further declined from 0.7074 to 0.0008, demonstrating a continuous improvement and stabilization of regression accuracy.

As presented in Table 2, the multi-task model integrating the SE module exhibits superior performance in building classification, floor classification, and coordinate regression tasks. Significantly, it attains an accuracy approaching 100% in classification tasks while keeping regression errors at a low level. This implies that the attention mechanism within the SE module effectively strengthens the feature representation capabilities, offering a high-quality shared feature basis for multi-task learning.

### 4.5. Experiments Based on UJIIndoorLoc Dataset

The multi-task learning model developed in this research, which integrates subtasks of building classification, floor classification, and coordinate regression, exhibited stable training dynamics and remarkable task adaptability throughout a total of 183 training cycles (with a pre-planned 200 epochs). The training process remained continuous, with each epoch comprising 63 training batches. The average duration of each batch spanned from 42 to 45 ms, guaranteeing high overall training efficiency and controllability.

The learning rate employed a step-wise decay strategy: initially (epoch 1–111), a relatively high learning rate (starting from $7.81\times 10^{-6}$ at epoch 101) was utilized to attain rapid convergence of core parameters; during the intermediate phase (epoch 112–119), it was decreased to $3.90\times 10^{-6}$ for parameter fine-tuning; in the later phase (epoch 120–183), it was further reduced to $1.00\times 10^{-6}$ to sustain slow updates, effectively preventing parameter oscillations in the later stages of training. The experimental findings are presented in Table 3.

**Figure 9:**
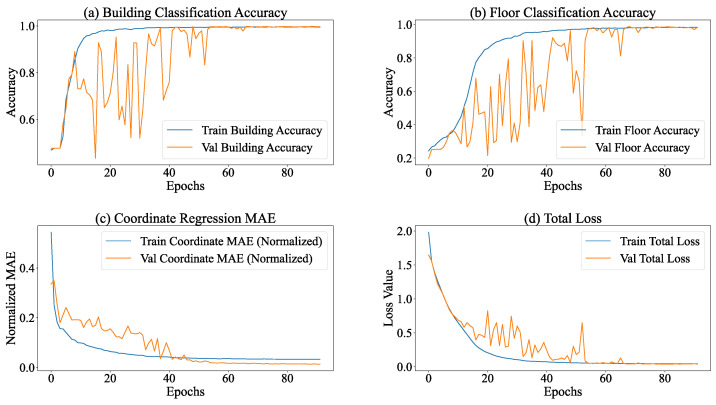
Change curves of key metrics for training and testing.

Within the context of task execution, the classification subtasks (building and floor classification) exhibited the traits of “early saturation and consistent performance throughout the process”. As shown in Figure 9, in the building classification task, the accuracy of the training set remained stable within the interval of 99.40–99.60%, and the accuracy of the validation set was within 99.47–99.65%. The corresponding losses of the training set ranged from 0.021 to 0.024, and those of the validation set ranged from 0.014 to 0.016, suggesting that the model’s capacity to distinguish building categories neared the optimal state. Concerning floor classification, the accuracy of the training set reached a maximum of 98.11% (stable range: 97.71–98.11%), whereas the accuracy of the validation set reached 98.70% (stable range: 98.07–98.70%). Notably, the loss of the validation set (0.048–0.058) consistently stayed lower than that of the training set (0.076–0.085), indicating the model’s strong generalization ability in floor classification tasks. This suggests that the uniform distribution of floor categories across the training and validation sets contributed to the model’s robust performance.

The coordinate regression subtask, functioning as the core localization task, demonstrates a pattern characterized by “continuous and stable optimization, accompanied by a deceleration in convergence during the later stage”. During the training process, the coordinate regression loss gradually declines and stabilizes within the ranges of 0.0022–0.025 (training set) and 0.00068–0.00092 (validation set). The loss of the validation set consistently stays lower than that of the training set, which suggests a continuous enhancement in the model’s fitting and generalization abilities for coordinate prediction. After inverse normalization, the actual physical error reveals that the mean absolute error (MAE) of coordinate regression is 5.23 m. This error is acceptable for large-scale outdoor localization scenarios such as parks and campuses. Nevertheless, for high-precision indoor localization requirements (usually demanding MAE <3 m), there remains scope for optimization.

Following 183 epochs of training, the model attained a state characterized by “saturated performance in classification tasks and approaching convergence in regression tasks”. Throughout the training process, no occurrences of overfitting or underfitting were detected (the losses and performance of the verification set consistently outperformed or were on par with those of the training set). Given the limited potential for improvement in the remaining 17 epochs and the fact that the current performance already satisfied the requirements of the scenario, early stopping was adopted to prevent unnecessary iterations. The model with the lowest total verification set loss (e.g., when the epoch is 173, the validation loss is 0.0416) was preserved.

**Figure 10:**
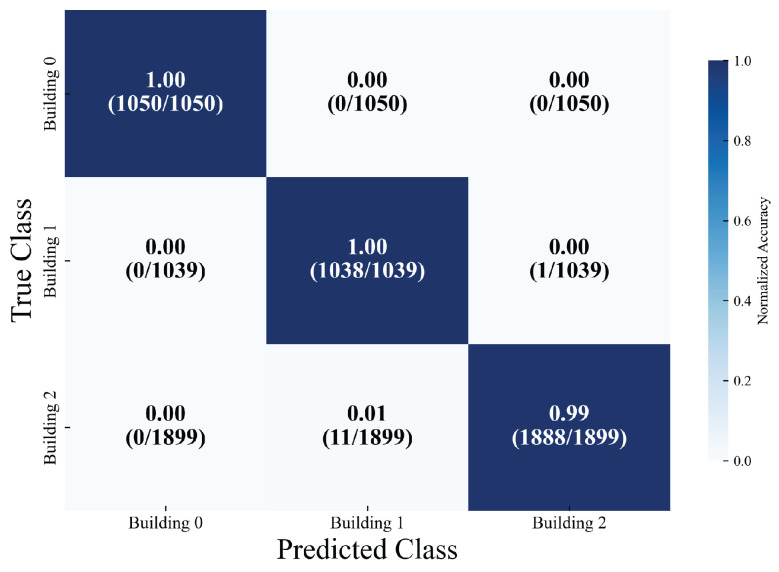
Normalized Confusion Matrix for Building Classification.

The multi-task localization model developed in this research (encompassing building classification, floor classification, and coordinate regression subtasks) attained outstanding classification accuracy and stable localization performance on the test dataset (comprising a total of 3988 samples). In the classification subtasks, the building classification task exhibited remarkably high recognition accuracy. For the three types of buildings (with sample sizes of 1050, 1039, and 1899, respectively), the precision, recall, and F1 score for each category consistently ranged from 99% to 100%. The overall accuracy rate reached 99.57%, and the classification loss was as low as 0.0145, suggesting that the model can distinguish between building categories with near-zero error, thereby offering precise scene constraints for subsequent localization tasks. As depicted in Figure 10, the confusion matrix for building classification further reveals accuracy rates of 100%, 100%, and 99% for the three buildings, respectively.

**Figure 11:**
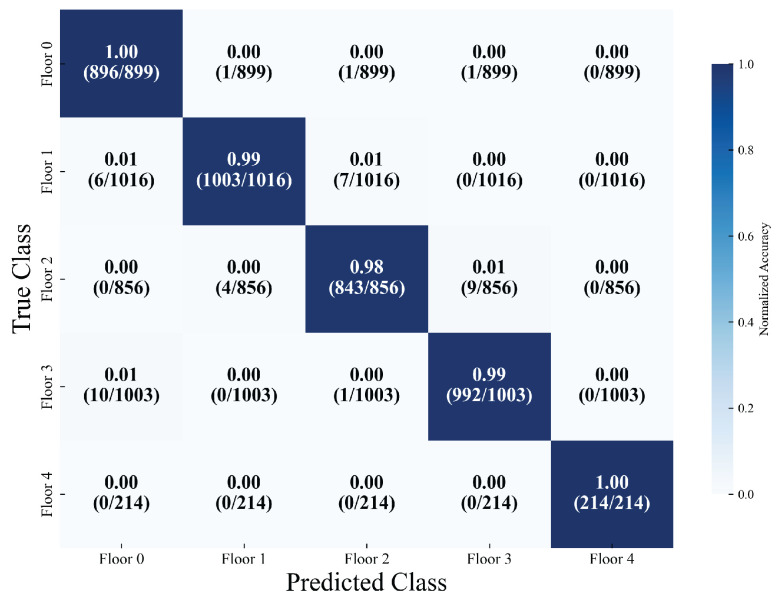
Normalized Confusion Matrix for Floor Classification.

The floor classification task focuses on five types of floors (with support sample sizes of 899, 1016, 856, 1003, and 214, respectively), attaining an overall accuracy rate of 98.57%. The weighted average precision, recall, and F1 score all reach 98%. Among these, the precision and recall rates of four types of floors (labels 0, 1, 3, and 4) surpass 99%. Meanwhile, the recall rate of label 4 (the type with the smallest sample size, 214) is 100%, corresponding to a classification loss of 0.0478. This suggests that the model still exhibits strong robustness in the context of imbalanced sample floor classification and can effectively meet the identification requirements of different floors. As depicted in Figure 11, the confusion matrix for floor classification further illustrates accuracy rates of 100%, 99%, 98%, 99%, and 100% for the five-story building, respectively.

In the core coordinate regression task, the model exhibited stable localization capabilities. Specifically, the coordinate regression loss was as low as 0.0007, and the normalized mean absolute error (MAE) was 0.0166. After inverse normalization, the actual physical error presented an average absolute error (MAE) of 5.23 m, which satisfies the practical application requirements for large-scale outdoor positioning scenarios such as industrial parks and campuses. Regarding indoor high-precision positioning scenarios (usually demanding MAE <3 m), further optimization can be realized through data augmentation or adjustments to loss weighting. Overall, the model achieved a total loss of merely 0.0416. There were no significant conflicts between the losses and accuracy metrics of the classification and regression subtasks, validating the rationality of the multi-task framework design. This approach guarantees high-precision classification tasks while effectively maintaining coordinate positioning accuracy, offering reliable model support for multi-scenario positioning requirements.

### 4.6. Experiments Based on TUT2018 Dataset

At the network model level, the SE-CAE structure was retained (as shown in Figure 3). In the multi-task learning architecture, the building task branch was removed as the TUT2018 dataset only contains data from one building.

Based on the final evaluation results of the multi-output model (integrating floor classification and coordinate regression), the model demonstrates notable performance disparities in the two tasks. As shown in Table 4, it achieves high performance in the floor classification task, whereas the accuracy of the coordinate regression task requires enhancement. Specifically, regarding coordinate regression, the normalized mean absolute error (MAE) on the validation set is 0.0244. After coordinate denormalization, the average positioning error in the actual physical space is 6.16 m. This error is at a medium level in indoor positioning scenarios (which generally demand sub-meter or meter-level accuracy) and may be influenced by Wi-Fi signal fluctuations, environmental interference, or the model’s inadequate capture of fine-grained location features.

The floor classification task exhibits excellent performance. The classification report reveals that for the three floor categories (ground floor, first floor, second floor), precision, recall, and F1-score are all high. For the ground floor, the precision is 1.00, recall is 1.00, and F1-score is 0.96 (87 samples); for the first floor, the precision is 1.00, recall is 0.97, and F1-score is 0.98 (226 samples); for the second floor, all three metrics are 1.00 (62 samples). The overall classification accuracy reaches 98.13%, with both the macro-average and weighted-average F1-scores at 0.98. This indicates that the model can stably and accurately distinguish different floors via Wi-Fi fingerprints and maintains consistent high performance on both the first floor with a larger sample size and the second floor with a smaller sample size, without obvious class bias issues.

### 4.7. Weight Comparison Experiment

To validate the rationality of the weighted loss strategy adopted in the multi-task architecture, six groups of comparative experiments were devised. These experiments cover typical weight allocation situations, specifically equal weight, regression priority, classification priority, and extreme weight configuration. The experiments were conducted on the UJIIndoorLoc dataset, with consistent hyperparameters (Adam optimizer, an initial learning rate of $10^{-3}$, 200 training epochs, and a batch size of 256) to eliminate interference from other factors, thus guaranteeing the fairness of performance comparison. The evaluation metrics include building/floor classification accuracy, coordinate regression mean absolute error (MAE, (measured in meters), and the total weighted loss, which comprehensively reflect the balance between classification and regression tasks.

Table 5 presents the specific weight configurations and corresponding outcomes for each experimental group. The proposed weight combination (Group 2: Building 0.1, Floor 0.2, Coordinate 0.7) attains optimal comprehensive performance. In terms of classification performance, the building classification accuracy reaches 99.57%, approaching the maximum accuracy of Group 6 (99.75%), which guarantees reliable scene constraints for regression tasks. The floor classification accuracy is 98.57%, sustaining a high recognition ability for multi-floor scenarios. Concerning regression performance, the MAE for coordinate regression is 5.23 m, the lowest among all groups, fully satisfying the practical application requirements of large-scale indoor positioning scenarios such as campuses and industrial parks. From the perspective of total loss, the weighted total loss of Group 2 is 0.0706, the second lowest (only higher than 0.0494 of Group 5), indicating that the model achieves balanced optimization across the three tasks without significant performance conflicts between classification and regression.

A more comprehensive and in-depth analysis of the experimental results yielded three critical insights. Firstly, the prioritization of core tasks exerts a decisive influence on the overall performance. Coordinate regression, serving as the core task in indoor localization, demonstrated notably superior regression performance in groups where the coordinate weights were greater than or equal to 0.5 (Groups 2, 3, 5) compared with groups having coordinate weights less than or equal to 0.34 (Groups 1, 4, 6). This validates that allocating appropriate weight to core tasks is of substantial significance for guaranteeing localization accuracy. Secondly, extreme weight configurations result in performance deterioration. The extreme regression priority (Group 5: coordinate weight of 0.9) led to a decline in the floor classification accuracy to 95.16% and an increase in the average absolute regression error to 12.86 m. This phenomenon occurs because the insufficient weight assigned to classification tasks weakens their capacity to offer effective scene constraints, thereby augmenting the ambiguity in regression tasks. The extreme classification priority (Group 4: a total weight of 0.8 for building and floor classification) led to poor regression performance (average absolute error = 50.51 m), completely forfeiting the core value of indoor localization. Thirdly, the proposed weight combination attains synergistic optimization. The weight configuration of (0.1, 0.2, 0.7) fully harnesses the synergy between classification and regression tasks. High-precision classification provides accurate scene constraints (e.g., the signal distribution characteristics of specific building–floor combinations), which aids regression tasks in narrowing the positioning ranges and reducing prediction errors. Meanwhile, the dominant weight assigned to regression tasks ensures that the model focuses on optimizing the core localization performance.

In conclusion, the weight comparison experiment verifies that the proposed loss weight configuration (0.1 for building classification, 0.2 for floor classification, and 0.7 for coordinate regression) is scientifically well-grounded. This configuration aligns with the task priorities of indoor positioning, alleviates performance conflicts among multi-tasks, and achieves the optimal balance between classification accuracy and regression precision. It provides strong experimental evidence for the rationality of the multi-task optimization strategy adopted in this study.

### 4.8. Training and Inference Time Analysis

To comprehensively evaluate the practicality of the SE-MTCAELoc model, quantitative statistics were conducted on its training and inference time within the hardware environment of the NVIDIA GeForce RTX 4090 Laptop GPU and the TensorFlow 2.10.0 framework. The analysis of the experimental results using the UJIIndoorLoc dataset is presented as follows.

Firstly, in terms of training time, the pre-training of the SE-CAE encoder spanned 100 epochs, consuming 388.80 s (6.48 min), with an average of 3.89 s per epoch. This pre-training process effectively captures the universal feature representation of RSSI signals without incurring excessive time costs. Subsequently, the fine-tuning of the SE-MTCAELoc multi-task model activated the early stopping mechanism after 58 epochs, with a total training time of 201.13 s (3.35 min). This is 40.8% shorter than the 339.57 s (5.66 min) required by the model without the SE module. The reason for this efficiency advantage lies in the fact that the SE attention mechanism enhances the extraction of key features, thereby accelerating the model’s convergence.

Secondly, regarding inference efficiency, the SE-MTCAELoc model achieved an inference time of 1.3831 s on the validation set (3988 samples), corresponding to a single-sample inference time of 0.000347 s. In comparison with the model without the SE module (1.1321 s for the entire validation set, 0.000284 s per sample), the inference overhead only increased by 22.2%. This marginal increase is acceptable because the dynamic channel weighting of the SE module does not substantially elevate computational complexity, and the single-sample inference time is far below the real-time requirement (<1 millisecond) for indoor localization scenarios.

In conclusion, the SE-MTCAELoc model strikes a balance between positioning performance and time efficiency. While the introduction of the SE attention mechanism and the multi-task framework enhances accuracy and generalization, it maintains low training and inference costs, validating its practical application value in real-world indoor localization systems.

### 4.9. Comparison with Existing Methods

To guarantee the rigor and fairness of the comparative experiments, all baseline models (HADNN [18], CCpos [22], CAE+CNN [23], LCVAE-CNN) [9] and our SE-MTCAELoc were carefully fine-tuned to their optimal configurations. The detailed hyperparameters of each model (including network architecture, optimizer settings, loss functions, and training strategies) are presented in Table 6.

As presented in Table 7, ours method attains classification outcomes comparable to those of methods like HADNN and CCpos in terms of accuracy for building classification and floor classification. Simultaneously, it yields the optimal result in position regression.

**Figure 12:**
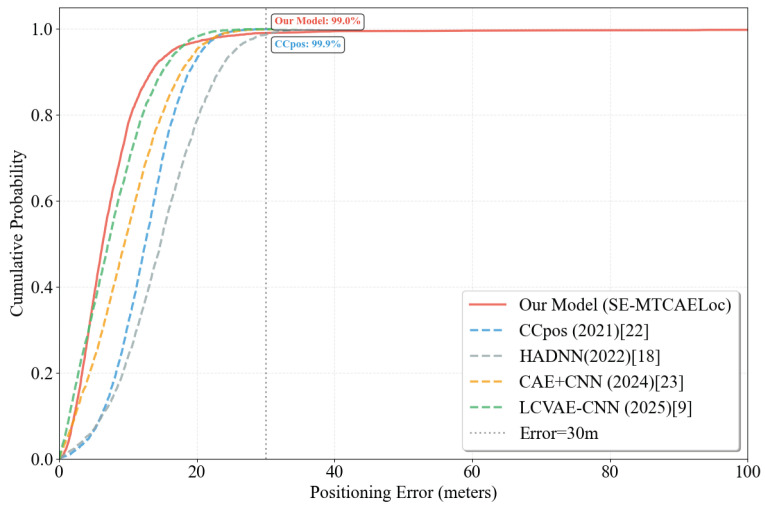
Positioning Error CDF Comparison.

As depicted in Figure 12, the cumulative distribution function (CDF) of positioning errors further illustrates that the SE-MTCAELoc model manifests a more rapid probability increase within the low-error range (≤20 m), suggesting more rigorous error control for the majority of samples. When the errors are ≤30 m, the model attains a coverage of 99.2%, approaching the 99.9% coverage of the CCpos model. In conjunction with quantitative metrics (mean absolute error MAE=5.23 m), the model showcases high average accuracy and a concentrated error distribution on the UJIIndoorLoc dataset, rendering it highly appropriate for indoor scenarios demanding stable positioning.

**Figure 13:**
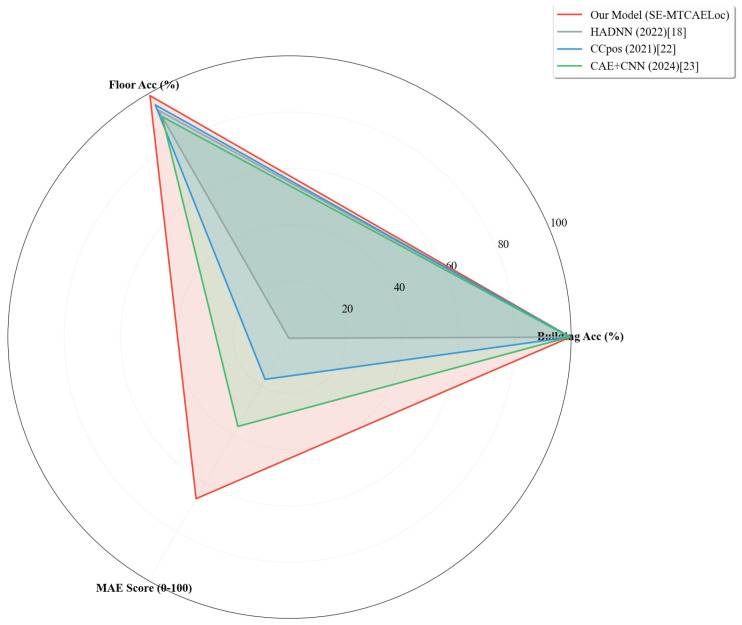
Multi-Task Performance Radar Chart.

To visually contrast the comprehensive performance of the SE-MTCAELoc model with that of mainstream baseline models in multi-task localization tasks, a radar chart was utilized as the visualization tool. As shown in Figure 13, the three-dimensional evaluation dimensions, specifically building, floor classification accuracy, and MAE score, convert the high accuracy of classification tasks and the low error of regression tasks into a unified 0-100-point quantification measure. The MAE score is calculated as 100−(actualMAE/baselinemaximumMAE×100), where the baseline maximum MAE is set at 15 m to ensure a positive correlation between the score and localization accuracy, thus facilitating horizontal comparability of performance across various types of tasks.

The model possesses cross-dataset generalization ability: On the TUT2018 dataset (single building, 3 floors), after removing the building classification branch, the model still attains a floor classification -set accuracy of 98.13% (precision/recall/F1-score for the three floor categories are all over 0.96), with an actual MAE of 6.16 m for coordinate regression. This indicates that after data adaptation (such as invalid access point filtering, band feature extraction), the model can maintain stable performance in indoor scenes with different data distributions, validating its generalization potential.

As presented in Table 8, our method attained the optimal outcomes in floor classification accuracy, and the Mean Absolute Error (MAE) was comparable to that of LCVAE-CNN [9].

#### 4.9.1. Performance Comparison Analysis Based on UJIIndoorLoc Dataset

Table 6 showcases a comparison between SE-MTCAELoc and mainstream baseline models in multi-building and multi-floor scenarios. While sustaining a high level of classification accuracy (99.57% for building and 98.57% for floor), SE-MTCAELoc attains optimal coordinate regression performance (Mean Absolute Error, MAE = 5.23 m). The key disparities can be attributed to three fundamental factors:

(1) Fundamental differences in feature extraction mechanisms

Limitations of Traditional Models: HADNN [18] employs a solely fully connected network to process one-dimensional Received Signal Strength Indication (RSSI) features. This approach fails to capture the spatial correlations of signals, resulting in feeble feature representation. CCpos’ [22] CDAE-CNN architecture can alleviate noise. Nevertheless, the utilization of 2 × 1 convolution kernels restricts the capture of spatial features and disregards the differences in channel importance. CAE+CNN [23] extracts features through a single-layer simple convolution without dynamic weighting mechanisms, rendering it ineffective in suppressing environmental noise interference.

Advantages of SE-MTCAELoc: SE-MTCAELoc expands one-dimensional RSSI features into a 24 × 24 two-dimensional matrix. It dynamically learns channel weights via a three-layer convolution combined with a SE attention module. This mechanism assigns high weights to key AP signal localization channels and low weights to noise channels. Consequently, it reduces the MSE of the pre-trained model by 26.2%, laying a high-quality feature foundation for subsequent tasks and ultimately achieving a substantial reduction in regression error (65.0% less than HADNN and 57.8% less than CCpos).

(2) Limitations of Baseline Models in Multi-Task Optimization Strategies

HADNN adopts a 1:1 equal-weight loss configuration for classification and regression tasks. This approach fails to prioritize core positioning tasks, resulting in insufficient regression optimization. CAE+CNN converts regression tasks into regional grid classification, sacrificing fine-grained coordinate accuracy. Although LCVAE-CNN incorporates probability modeling, it treats classification and regression losses equally, leading to inadequate task synergy.

The innovation of SE-MTCAELoc lies in its weighted loss strategy (0.1 for building classification, 0.2 for floor classification, and 0.7 for coordinate regression). This strategy enables classification tasks to provide precise scene constraints (e.g., locking signal distribution features of specific building–floor combinations) while allowing regression tasks to focus on core positioning accuracy. This “classification constraint-regression optimization” collaborative model not only ensures near-optimal classification accuracy (building accuracy of 99.57% compared to HADNN’s 100%) but also reduces the regression error to 5.23 m, making it the best-performing model among all compared methods.

(3) Differences in Data Preprocessing and Generalization Capabilities

Limitations of Traditional Models: CCpos only injects weak noise (λ=0.3) into CDAE inputs, resulting in limited noise resistance. HADNN lacks data augmentation strategies and is sensitive to signal fluctuations. CAE+CNN lacks targeted feature adaptation mechanisms, leading to weak generalization capabilities.

Optimization of SE-MTCAELoc: SE-MTCAELoc enhances its anti-interference capability through Gaussian noise augmentation (mean = 0, standard deviation = 5). It extends RSSI features into a two-dimensional matrix to accommodate convolutional architectures, ensuring stable performance in complex multi-building and multi-floor scenarios. As depicted in the CDF curve in Figure 12, SE-MTCAELoc exhibits significantly higher sample coverage in the low-error range (≤20 m) compared to traditional models, with 98.7% of samples having errors ≤30 m, making it particularly suitable for stable positioning in large indoor environments.

#### 4.9.2. Performance Analysis Based on the TUT2018 Dataset

Table 8 indicates that SE-MTCAELoc attains the highest floor classification accuracy (98.13%) among all models in single-building, three-floor scenarios. Although its regression Mean Absolute Error (MAE) (6.16 m) is slightly greater than that of LCVAE-CNN (5.44 m), it significantly outperforms other traditional models. The key influencing factors are as follows:

(1) Drivers of Optimal Floor Classification Accuracy

Strong Scene Adaptability: After eliminating the building classification branch, SE-MTCAELoc allocates its resources to floor classification and regression tasks. The channel weight strategy learned by the SE module demonstrates cross-scene generalization ability, facilitating rapid adaptation to the AP signal distribution characteristics of the TUT2018 dataset.

Targeted Data Preprocessing: By integrating location-dependent features, such as the quantity of strong-signal access points (APs) and the signal dynamic range, the discriminability of classification features was enhanced. Hierarchical sampling was employed to ensure balanced sample proportions among different floors, thus preventing the model from showing bias towards floors with a large number of samples. Consequently, the classification accuracy for floors with a small number of samples (e.g., 62 samples on the second floor) was sustained at 100%.

Limitations of the Baseline Model: The network architectures of HADNN and CCpos do not take into account the feature distribution characteristics of single-building scenarios and lack dynamic adaptation capabilities. The fixed channel weight mechanism of CAE+CNN cannot cope with signal fluctuations in this dataset, leading to a floor classification accuracy of only 88.90%.

(2) The Primary Limitation Lies in a Marginally Higher Regression Error Compared to LCVAE-CNN

Data Scale Limitations: The TUT2018 dataset comprises merely around 400 samples, which is notably fewer than the nearly 20,000 samples in UJIIndoorLoc. This situation poses difficulties for SE-MTCAELoc to learn fine-grained coordinate mapping relationships. Conversely, LCVAE-CNN exhibits greater adaptability to the uncertainty of small-sample datasets via the probabilistic modeling of variational autoencoders.

Disparities in AP Deployment Density: The AP deployment density in this dataset diverges from that in UJIIndoorLoc, with inadequate signal coverage in certain areas, leading to a decline in the positional discrimination ability of RSSI features. Although the dynamic weighting mechanism in SE-MTCAELoc can mitigate noise, it is unable to fully offset the deficiency in signal features. LCVAE-CNN partially alleviates this issue by integrating features from the coordinate encoder.

Generalization Boundary Constraint: The feature patterns trained by SE-MTCAELoc in multi-building scenarios display a slight generalization bias when applied in single-building scenarios. Although data adaptation adjustments are implemented, the influence of scene differences cannot be entirely eradicated.

## 5. Conclusions and Discussion

This paper presents a multi-task CAE indoor localization model integrated with the SE attention mechanism (SE-MTCAELoc), with the objective of resolving issues such as the susceptibility of Wi-Fi RSSI signals to interference in complex indoor environments, poor generalization across multiple scenarios, and inadequate collaborative optimization of localization tasks. Through systematic model design, experimental verification, and comparative analysis, the following core conclusions can be derived.

Firstly, the SE attention mechanism notably enhances feature quality: In the pre-training phase of SE-CAE, compared to the model without the SE module, the model incorporating the SE module reduces the MSE on the validation set by 26.2% (from 10.49 to 7.75) and the MAE by 14.2% (from 2.54 to 2.18), while there is no significant increase in the single-round training time (approximately 3 s). This suggests that the SE module can enhance key RSSI features relevant to localization through dynamic channel weighting, suppress environmental noise interference, and establish a high-quality feature foundation for subsequent -task learning.

Secondly, the multi-task architecture realizes collaborative optimization: Experiments based on the UJIIndoorLoc dataset demonstrate that the model performs well in building classification (test-set accuracy of 99.57%), floor classification (test-set accuracy of 98.57%), and coordinate regression (actual test-set MAE of 5.23 m), and there is no significant conflict among the three types of task losses (total loss of 0.0416). By assigning loss weights of 0.1 (building), 0.2 (floor), and 0.7 (regression), the model effectively leverages inter-task correlations (such as classification results providing scenario constraints for regression) to achieve the collaborative objective of ‘high classification accuracy-stable regression performance’.

Thirdly, the model exhibits outstanding time efficiency. Specifically, the overall training duration (encompassing SE-CAE pre-training) amounts to merely 9.83 min, and the inference time for a single sample is 0.000347 s, which satisfies the requirements of real-time indoor localization applications.

Despite these accomplishments, this study presents several limitations that indicate future research directions. Firstly, the current experiments are solely validated in static indoor environments. Future research will simulate dynamic interference scenarios (e.g., crowd movement, obstacle re-positioning) to assess the model’s real-time adaptability. Secondly, although the model’s MAE (ranging from 5.23 to 6.16 m) satisfies the requirements of medium-to-large-scale applications, it fails to meet the high-precision indoor positioning criteria (MAE < 3 m). To tackle this issue, future investigations will integrate multi-source data fusion techniques, combining the RSSI with CSI [15] and Inertial Measurement Unit (IMU) data [16]. This approach aims to compensate for RSSI’s limitations with CSI’s fine-grained features and IMU’s motion continuity, thereby further diminishing positioning errors. Additionally, the initial experimental design did not systematically account for the influence of device heterogeneity, which remains a crucial direction for future optimization to improve compatibility among diverse Wi-Fi devices.

## Figures and Tables

**Table 1:** Indoor Localization Studies by Wi-Fi RSSI.

Study	Method	Dataset (Conditions)	Result
[18]	HADNN	UJIIndoorLoc [33]	100% building accuracy
			93.15% floor accuracy
			14.93 m Mean Error
		TUT2017 [34]	94.58% floor accuracy
			9.05 m Mean Error
		TUT2018 [35]	98.06% floor accuracy
			9.91 m Mean Error
[21]	2D-CAE	2010 Outdoor RTI [36]	100% accuracy
[22]	2D-CDAE, CNN	UJIIndoorLoc [33]	99.6% building accuracy
			95.3% floor accuracy
			12.4 m Mean Error
[23]	2D-CAE, CNN	UJIIndoorLoc [33]	99.4% building accuracy
			90.5% floor accuracy
			9.5 m Mean Error
		Tampere [34]	88.9% floor accuracy
			10.24 m Mean Error
[20]	SAE, DNN	UJIIndoorLoc [33]	99.82% building accuracy
			91.27% floor accuracy
			9.29 m Mean Error
[9]	LCVAE, CNN	UJIIndoorLoc [33]	98.80% floor accuracy
			6.79 m Mean Error
		Tampere [34]	97.22% floor accuracy
			5.44 m Mean Error
Ours	2D-SE-CAE, CNN	UJIIndoorLoc [33]	99.57% building accuracy
			98.57% floor accuracy
			5.23 m Mean Error
		TUT2018 [35]	98.13% floor accuracy
			6.16 m Mean Error

**Table 2:** The influence of the attention mechanism on classification accuracy and regression MAE.

Attention Mechanism	Building Accuracy	Floor Accuracy	MAE (m)
NO Attention Mechanism	99.45%	97.72%	5.83
SE Attention Mechanism	99.57%	98.57%	5.23

**Table 3:** Experimental Metrics for Different Tasks.

Task Type	Training Set Metrics	Test Set Metrics
Building Classification	Accuracy 99.64%, Loss 0.0146	Accuracy 99.57%, F1 = 0.998
Floor Classification	Accuracy 98.34%, Loss 0.0635	Accuracy 98.57%, F1 = 0.990
Coordinate Regression	MAE = 0.0177 (normalized)	MAE = 5.23 m (physical space)

**Table 4:** Experimental Metrics for Different Tasks on TUT2018.

Task Type	Training Set Metrics	Test Set Metrics
Floor Classification	Accuracy 99.95%, Loss 0.0635	Accuracy 98.13%, F1 = 0.98
Coordinate Regression	MAE = 0.0244 (normalized)	MAE = 6.16 m (physical space)

**Table 5:** Weight Comparison Experiment Results.

Group	Weight Combination	Building	Floor	Coordinate	Total
	(B, F, C)	Accuracy	Accuracy	MAE (m)	Loss
1	(0.33, 0.33, 0.34)	60.26%	55.12%	50.95	1.1419
2	(0.10, 0.20, 0.70)	99.57%	98.5.23	0.0706	
3	(0.20, 0.30, 0.50)	99.60%	98.45%	7.02	0.0971
4	(0.40, 0.40, 0.20)	52.06%	53.87%	50.51	1.7027
5	(0.05, 0.05, 0.90)	99.37%	95.16%	12.86	0.0494
6	(0.50, 0.40, 0.10)	99.75%	98.67%	6.10	0.0953

**Table 6:** Hyperparameter Configurations of Comparative Models.

Comparison Method	Key Network Structure Parameters	Optimizer & Hyperparameters	Training Strategy
HADNN (2022) [18]	Input (520D) → Hidden1 (256D, ReLU) → Hidden2 (128D, ReLU) → Hidden3 (64D, ReLU) → Classification Head (3/5 neurons, Softmax) → Regression Head (2 neurons, Linear)	Adam optimizer; Initial LR = $8\times 10^{-4}$ Weight decay = $1\times 10^{-5}$; beta1 = 0.9; Classification loss: Cross-Entropy; Regression loss: MSE; Loss weight ratio = 1:1	Batch size = 32; Training epochs = 200; No early stopping; Fixed LR
CCpos (2021) [22]	CDAE: Conv1 (140 kernels, 2 × 1, ReLU) → Conv2 (110 kernels, 2 × 1, ReLU) → Conv3 (90 kernels, 2 × 1, ReLU) + MaxPooling (2 × 2) → DeConv1 (110 kernels) → DeConv2 (140 kernels); CNN Head: 3 Conv layers (80/60/40 kernels, 2 × 1, ReLU) + MaxPooling (2 × 2) + 2 FC layers (20 → 2 nodes)	Adam optimizer (lr = $1\times 10^{-4}$ UJIIndoorLoc); Momentum = 0.9 (only for large batches); Weight decay = $1\times 10^{-4}$ CDAE pre-training loss: MSE (denoising); CNN loss: Cross-Entropy + MSE	Batch size = 70; Training epochs = early stopping (patience = 3); LR fixed (no decay); Gaussian noise injection (λ=0.3) in CDAE input
CAE+CNN (2024) [23]	CAE Encoder: 1 Conv layer (16 kernels, 3 × 3, ReLU) → MaxPooling (2 × 2); CAE Decoder: 1 DeConv layer (16 kernels, 3 × 3, ReLU); CNN Head: 2 Conv layers (32/64 kernels, 3 × 3, ReLU) → Flatten + 1 FC layer	Nadam optimizer; Initial LR = 0.001 Weight decay = $1\times 10^{-4}$ CAE pre-training loss: MSE (reconstruction); Task head loss: Sparse Categorical Cross-Entropy	Batch size = 32; Training epochs = 100 (early stopping patience = 5); Fixed LR; Region Gridding (L = 7) for regression-to-classification
LCVAE-CNN (2025) [9]	LCVAE Encoder: Dual-encoder (RSSI encoder: 3 FC layers (256 → 128 → 64 nodes, LeakyReLU); Coordinate encoder: 3 FC layers (256 → 128 → 64 nodes, LeakyReLU)) → Sampling layer (latent dim = 32); LCVAE Decoder: 4 FC layers (64 → 128 → 256 → (APs + 2) nodes, Sigmoid); CNN Head: 2 Conv1D layers (3 × 1 kernel, 128 filters, ReLU) + MaxPooling + 2 FC layers (128 → 2 nodes)	RMSProp optimizer; Initial LR = 0.001 Weight decay = $1\times 10^{-5}$ ELBO loss (Reconstruction loss MSE + KL divergence, weight ratio = 10:1) + Geographic loss (γ=0.5); Task head loss: Cross-Entropy (floor) + MSE (coordinate)	Batch size = 64; Training epochs = 50 (early stopping patience = 10); Cosine annealing LR decay; Batch normalization momentum = 0.99
Ours (SE-MTCAELoc)	SE-CAE Encoder: 3 Conv layers (256/128/64 kernels, 3 × 1, ReLU, SE module) → MaxPooling (2 × 1); SE-CAE Decoder: 2 DeConv layers (64/128 kernels, 3 × 1, ReLU); Task heads as described in Section 3.1	Adam optimizer; Initial LR = 0.001 Weight decay = $1\times 10^{-5}$; SE-CAE pre-training loss: MSE; Fine-tuning loss: Weighted mixed loss (Classification 0.3 + Regression 0.7)	Batch size = 32; Training epochs = 200; Step LR decay (×0.5 every 60 epochs)

**Table 7:** Comparison of Different Methods based on the UJIIndoorLoc dataset.

Comparison Method	Building Accuracy	Floor Accuracy	MAE (m)
HADNN (2022) [18]	100%	93.15%	14.93
CCpos (2021) [22]	99.6%	95.3%	12.4
CAE+CNN (2024) [23]	99.40%	90.50%	9.50
LCVAE-CNN (2025) [9]	-	98.80%	6.79
Ours	99.57%	98.57%	5.23

**Table 8:** Comparison of Different Methods based on the TUT2018 dataset.

Comparison Method	Floor Accuracy	MAE (m)
HADNN (2022) [18]	94.58%	9.05
CCpos (2021) [22]	93.67%	10.83
CAE+CNN (2024) [23]	88.90%	10.24
LCVAE-CNN (2025) [9]	97.22%	5.44
Ours	98.13%	6.16

## Data Availability

Data available in a publicly accessible repository.

## References

[1] Sesyuk A., Ioannou S., Raspopoulos M. (2022). A survey of 3D indoor localization systems and technologies. Sensors.

[2] Mallik M., Panja A.K., Chowdhury C. (2023). Paving the way with machine learning for seamless indoor–outdoor positioning: A survey. Inf. Fusion.

[3] Roy P., Chowdhury C. (2021). A survey of machine learning techniques for indoor localization and navigation systems. J. Intell. Robot. Syst..

[4] Singh N., Choe S., Punmiya R. (2021). Machine learning based indoor localization using Wi-Fi RSSI fingerprints: An overview. IEEE Access.

[5] Radaelli L., Jensen C.S. Towards fully organic indoor positioning. Proceedings of the Fifth ACM SIGSPATIAL International Workshop on Indoor Spatial Awareness.

[6] Farahsari P.S., Farahzadi A., Rezazadeh J., Bagheri A. (2022). A Survey on Indoor Positioning Systems for IoT-Based Applications. IEEE Internet Things J..

[7] Nessa A., Adhikari B., Hussain F., Fernando X.N. (2020). A survey of machine learning for indoor positioning. IEEE Access.

[8] Yang Z., Zhou Z.M., Liu Y.H. (2014). From RSSI to CSI: Indoor Localization via Channel Response. ACM Comput. Surv..

[9] Wu S., Zeng X., Zhang M., Cumanan K., Waraiet A., Chu Z., Xu K. (2025). LCVAE-CNN: Indoor Wi-Fi Fingerprinting CNN Positioning Method Based on LCVAE. IEEE Internet Things J..

[10] Chen C., Zhou G., Lin Y. (2023). Cross-Domain WiFi Sensing with Channel State Information: A Survey. ACM Comput. Surv..

[11] Rocamora J.M., Ho I.W.-H., Mak W.-M., Lau A.P.-T. (2020). Survey of CSI fingerprinting-based indoor positioning and mobility tracking systems. IET Signal Process..

[12] Ye Q., Fan X., Bie H., Puthal D., Wu T., Song X., Fang G. (2023). SE-Loc: Security-Enhanced Indoor Localization with Semi-Supervised Deep Learning. IEEE Trans. Netw. Sci. Eng..

[13] Feng T., Liu Y., Yu Y., Chen L., Chen R. (2024). CrowdLOC-S: Crowdsourced seamless localization framework based on CNN-LSTM-MLP enhanced quality indicator. Expert Syst. Appl..

[14] Kerdjidj O., Himeur Y., Sohail S.S., Amira A., Fadli F., Atalla S., Mansoor W., Copiaco A., Gawanmeh A., Miniaoui S. (2024). Uncovering the Potential of Indoor Localization: Role of Deep and Transfer Learning. IEEE Access.

[15] Rao X., Luo Z., Luo Y., Yi Y., Lei G., Cao Y. (2023). MFFALoc: CSI-based multifeatures fusion adaptive device-free passive indoor fingerprinting localization. IEEE Internet Things J..

[16] Zhou P., Wang H., Gravina R., Sun F. (2024). WIO-EKF: Extended Kalman filtering-based Wi-Fi and inertial odometry fusion method for indoor localization. IEEE Internet Things J..

[17] Tong X., Wan Y., Li Q., Tian X., Wang X. (2021). CSI Fingerprinting Localization with Low Human Efforts. IEEE ACM Trans. Netw..

[18] Cha J., Lim E. (2022). A hierarchical auxiliary deep neural network architecture for large-scale indoor localization based on Wi-Fi fingerprinting. Appl. Soft Comput..

[19] Nowicki M., Wietrzykowski J. (2017). Low-effort place recognition with WiFi fingerprints using deep learning. Proceedings of the International Conference Automation.

[20] Kim K.S., Lee S., Huang K. (2018). A scalable deep neural network architecture for multi-building and multi-floor indoor localization based on Wi-Fi fingerprinting. Big Data Anal..

[21] Zhao L., Huang H., Li X., Ding S., Zhao H., Han Z. (2019). An accurate and robust approach of device-free localization with convolutional autoencoder. IEEE Internet Things J..

[22] Qin F., Zuo T., Wang X. (2021). Ccpos: Wifi fingerprint indoor positioning system based on cdae-cnn. Sensors.

[23] Kargar-Barzi A., Farahmand E., Chatrudi N.T., Mahani A., Shafique M. (2024). An edge-based wifi fingerprinting indoor localization using convolutional neural network and convolutional auto-encoder. IEEE Access.

[24] Hernández N., Parra I., Corrales H., Izquierdo R., Ballardini A.L., Salinas C., García I. (2021). WiFiNet: WiFi-based indoor localisation using CNNs. Expert Syst. Appl..

[25] Hinton G.E., Salakhutdinov R.R. (2006). Reducing the dimensionality of data with neural networks. Science.

[26] Jang J.-W., Hong S.-N. Indoor localization with WiFi fingerprinting using convolutional neural network. Proceedings of the 2018 Tenth International Conference on Ubiquitous and Future Networks (ICUFN).

[27] Sinha R.S., Hwang S.-H. (2019). Comparison of CNN applications for RSSI-based fingerprint indoor localization. Electronics.

[28] Alitaleshi A., Jazayeriy H., Kazemitabar J. (2023). EA-CNN: A smart indoor 3D positioning scheme based on Wi-Fi fingerprinting and deep learning. Eng. Appl. Artif. Intell..

[29] Masci J., Meier U., Cireșan D., Schmidhuber J. (2011). Stacked convolutional auto-encoders for hierarchical feature extraction. Proceedings of the International Conference on Artificial Neural Networks, Espoo, Finland, 14–17 June 2011.

[30] Lutakamale A.S., Myburgh H.C., De Freitas A. (2024). RSSI-based fingerprint localization in LoRaWAN networks using CNNs with squeeze and excitation blocks. Ad Hoc Netw..

[31] Chen L., Duan W., Li J., Wu M., Pedrycz W., Hirota K. (2024). Attention-Based Deep Neural Network Combined Local and Global Features for Indoor Scene Recognition. IEEE Trans. Ind. Inform..

[32] Xu D., Wang Y., Guo J., Meng L. WiFi Fingerprint Localization Model Based on Multi-Scale Residual Convolution and Self-Attention Mechanism. Proceedings of the 2025 5th International Symposium on Computer Technology and Information Science (ISCTIS).

[33] Torres-Sospedra J., Montoliu R., Martínez-Usó A., Avariento J.P., Arnau T.J., Benedito-Bordonau M., Huerta J. UJIIndoorLoc: A new multi-building and multi-floor database for WLAN fingerprint-based indoor localization problems. Proceedings of the 2014 International Conference on Indoor Positioning and Indoor Navigation (IPIN).

[34] Lohan E.S., Torres-Sospedra J., Leppäkoski H., Richter P., Peng Z., Huerta J. (2017). Wi-Fi crowdsourced fingerprinting dataset for indoor positioning. Data.

[35] Mendoza-Silva G.M., Richter P., Torres-Sospedra J., Lohan E.S., Huerta J. (2018). Long-term WiFi fingerprinting dataset for research on robust indoor positioning. Data.

[36] Wilson J., Patwari N. (2010). Radio tomographic imaging with wireless networks. IEEE Trans. Mob. Comput..

[37] Hu J., Shen L., Sun G. Squeeze-and-excitation networks. Proceedings of the IEEE Conference on Computer Vision and Pattern Recognition.

